# Theoretical design of blockchain-based traceability for organic egg supply chains according to regulation (EU) 2018/848

**DOI:** 10.1371/journal.pone.0304791

**Published:** 2024-06-11

**Authors:** Florian Zülch, Martin Holle, Andreas Hofmann

**Affiliations:** 1 Fakultät Life Sciences, Department Ökotrophologie, Hochschule für Angewandte Wissenschaften, Hamburg, Germany; 2 Nationales Referenzzentrum für Authentische Lebensmittel, Max Rubner-Institut, Kulmbach, Germany; 3 Melbourne Veterinary School, Faculty of Science, The University of Melbourne, Parkville, Victoria, Australia; University of Doha for Science & Technology, QATAR

## Abstract

The use of blockchain technology to establish food traceability chains has the potential to provide transparent information of food stuffs along the entire supply chain and also aid in the documentation or even execution of official food control processes. Particularly in instances where analytical methodologies cannot provide definitive data for food control questions under study, the certificate-based approach of a traceability chain may offer a way of regulatory control for state authorities. Given the rising importance of organic produce and the high share of eggs among the organic produce in the European Union as well as the new EU regulation on organic products and labelling that came into force in 2022, we analyze here how the control of egg production type and marketing standards can be represented within a blockchain-based traceability chain such as to maximize the traceability in compliance with the current relevant EU regulations. Intended for the use by the official food control authorities, a traceability chain for organically produced eggs in the EU would need to be implemented as a permissioned blockchain, since only select entities are allowed to participate. By combining a proof of authority consensus mechanism with issuance of soulbound tokens, we effectively suggest a ‘proof of soulbound authority’ consensus process. The soulbound tokens are issued throughout the administrative chain from the European Commission down to the official food control authorities in individual member states that ultimately certify the control bodies for organic produce. Despite the general limitation of not providing unambiguous proof of the organic status of individual products, the concept discussed here offers advantages with respect to allocation of authority at EU level and therefore might have positive effects beyond the traceability chain.

## 1. Introduction

European Union (EU) legislation contains many legal standards for the integrity and control of organically produced food. Nevertheless, there have been repeated cases of food fraud in the past. In order to protect consumers as well as generally human, animal and plant health, the EU has set out common rules for official controls to enforce measures along the agri-food chain (Regulation (EU) 2017/625), specifically identifying organic production and labelling as an important area to be checked for compliance.

Whereas the investigation of food stuffs by purely analytical means is often possible for questions relating to specific contents (presence of valorizing components, absence of unwanted substances, etc), the review of claims relating to provenance or production methods is notoriously difficult to be addressed by experimental analytics alone. Recent activities to scrutinize the provenance of foods of that have been produced regionally or adhere to the rules of food production void of deforestration [1] highlight the importance of novel solutions for food control subjects that cannot be fully evaluated by purely experimental means.

Assured traceability and a concomitantly reduced risk of fraud is expected from mapping supply chains with blockchain technology [2]; however, the backing of food supply chains with blockchain applications is not prevalent as of today [3]. Building on its blockchain strategy [4], the European Commission is actively promoting the development of blockchain applications in the food sector to further increase transparency in the food process chain [5]. Specifically, with its European Blockchain Partnership, efforts are under way to build a European Blockchain Services Infrastructure [6].

The application of blockchain solutions in the agri-food sector has previously been explored academically in livestock farming [7], including cattle [8] and turkey [9, 10], as well as egg production [11]. Furthermore, applications such as the IBM Food Trust [12], SAP’s cloud enterprise with the Leonardo Blockchain [13] as well as OpenSC by WWF-Australia and BCG Digital Ventures [14] offer traceability applications for the food industry. A generic system for traceability in the agri-food supply chain based on blockchain technology with a clear focus on the end consumer has previously been proposed [15, 16].

In Germany, eggs are the foodstuff being produced by organic means most frequently, with organically produced eggs taking a share of 13% of the overall egg production [17]. Whereas European regulations stipulate requirements regarding the traceability of food of animal origin that assure authorities of documented information about a shipment of such food, there are no specifications as to a traceability chain for shell eggs [18, 19]. To date, some private quality and origin traceability systems are operating in Germany, including the International Featured Standards [20] which egg marketers and packers need to subscribe to as per requirement by German food retailers. In addition, there is the Association for Controlled Alternative Animal Husbandry (KAT) as well as the Respeggt Group both of which offer traceability systems for eggs from marketers that subscribe to these systems [21, 22].

Focusing on the technological and legal circumstances in Midwestern USA, Bumblauskas and colleagues [23] suggested a blockchain solution for the traceability of organic eggs based on a Hyperledger Sawtooth-based traceability chain [16] all the way through from production to the end consumer. Conceptually, this solution could be applied to European organic egg supply chains as well. However, with EU regulation (EU) 2018/848 [24] on organic products and labelling coming into force in 2022, supply chain traceability applications need to consider the current regulatory content for products from organic production. The new legislation applies whenever there are indications of organic production on the label, advertising or commercial documents of a product [25]. It is therefore necessary to examine how and to what extent the application of these new legal standards in combination with the structural prerequisites by the controlling bodies, organic industry labels and official food control authorities allows the establishment of a blockchain-based traceability chain.

Here, on the basis of current legislation within the EU, we analyze how the control of egg production type and marketing standards can be represented within a blockchain-based traceability chain so that traceability is as complete as possible (traceability to prevent food fraud and ensure food authenticity) and in compliance with the principles of the EU regulation on organic products and labelling.

## 2. Methodology

Given that this present research constitutes mainly meta-analyses of relevant concepts and literature, this section summarizes the assembled facts, existing prerequisites and technological components, respectively, emanating from these analyses.

In order to find the critical determinants of a traceability solution with the supply chain of organically produced eggs under the present European legislation, we first separated the overall research question into its major constituting domains, i.e. the overall legislative situation, the intricacies of the organic egg supply chain and the various aspects of blockchain technology. The bodies of facts as relevant to each one of those domains are summarized in the following sections.

The set factual elements obtained in this step constitutes the base upon which considerations about necessities and possibilities of a traceability solution were made and evaluated, using occasional references to Germany as an example. In our chosen approach, these considerations and evaluations constitute the outcome of the research and are thus summarized under Results and Discussion.

The information on European legislation assembled and reviewed as part of this research was obtained from the primary sources of European Union law [26] as well as the online resources by the publisher Behr’s [27].

### 2.1 EU legislation on the production and marketing of organic eggs

An appraisal with a view towards concrete implementation in an individual member state would need to consider the laws of that member state. However, for the present work, we have limited the analysis to the current EU legislation. In order to outline the theoretical structure of a blockchain-based traceability chain for eggs from organic production that complies with European standards, we considered regulation (EU) 2018/848, complemented by the implementing regulation (EU) 2021/279, as the key legal standard to be applied. This regulation was adopted by the European Parliament and the Council, and is in effect since 2022. However, there exist other, independent regulations by the European Commission that often deal with identical subjects [28].

**Fig 1:**
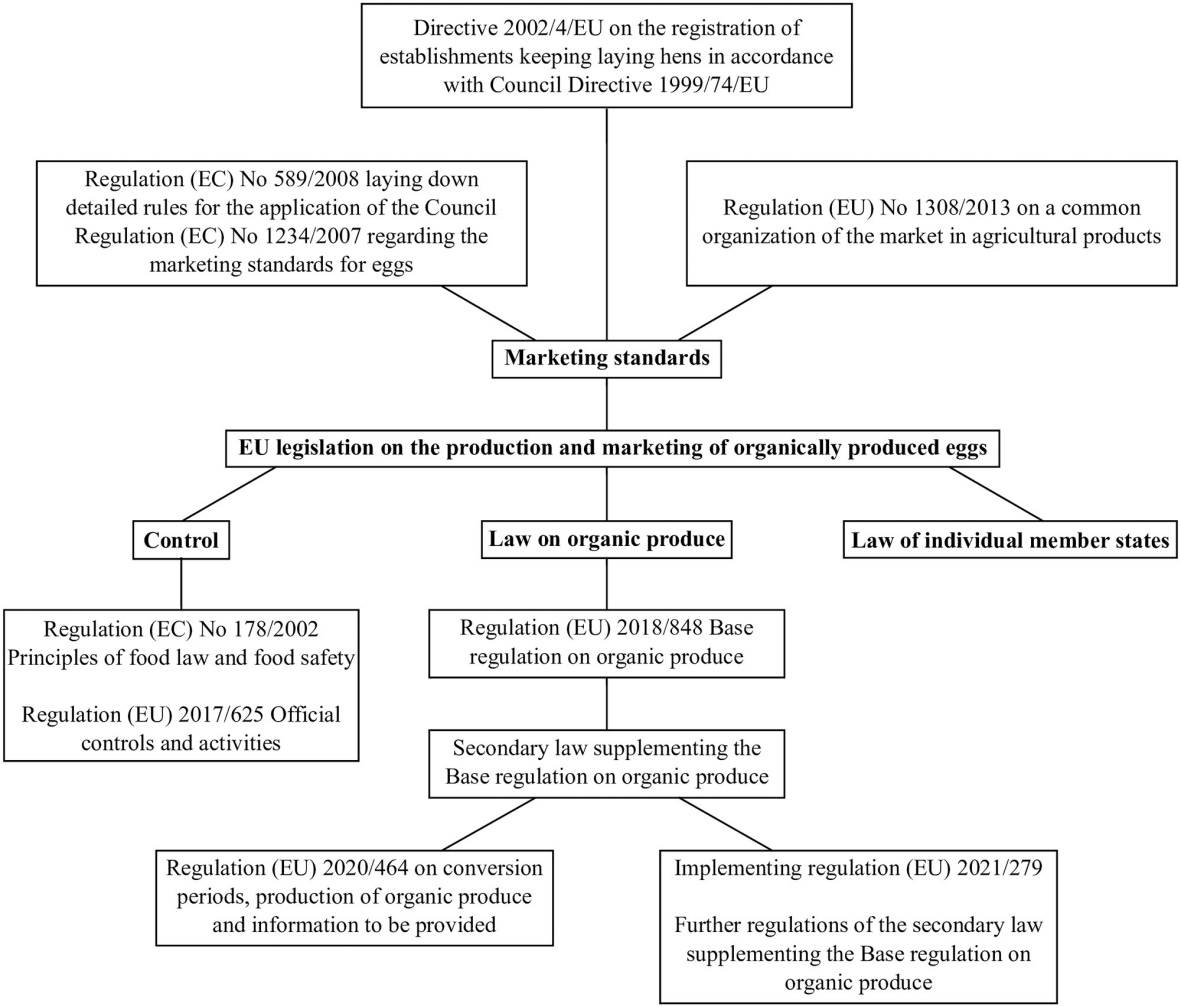
EU legislation relevant for production and marketing of organically produced eggs (for further details please see S1 Table).

Several regulations in the subject areas of production, control and third-country imports (imports from non-EU countries) can be understood as secondary legislation to (EU) 2018/848. Additionally, regulation (EU) 2017/625 sets out the requirements for the implementation of controls regarding organically produced food for all EU member states. Fig 1 summarizes the most relevant legislation in this context; for further details see S1 Table.

### 2.2 Specifications for the production and labelling of organic food

Regulation (EU) 2018/848, together with its implementing regulations, specify detailed and comprehensive requirements for the production of plant- and animal-derived food. These include (i) the renunciation of synthetic chemical pesticides and easily soluble mineral fertilizers, (ii) embedding of production processes into ecological circulation systems, (iii) the preservation and activation of the biological activity of agricultural soil areas, (iv) the control of pests, diseases and weeds by holistic methods, (v) land-based animal husbandry commensurate with species requirements, (vi) ecologically oriented feeding without the addition of antibiotics and performance-enhancing substances, and (vii) a general ban on the use of genetically modified organisms and products made from them [25].

When distributing the food to retailers and consumers, labelling of organic produce may occur by either one of three options. The primary organic label may either be given in the sales description of the food, or a component label may be shown only in the list of ingredients. A separate third option applies to products whose main ingredients derive from hunting or fishing [25]. If the labelling is included in the sales description (option 1), the use of the EU organic logo is mandatory. Additionally, national logos such as the German organic seal or logos of industry associations can be shown. Within the same field of vision of the EU organic logo, the place of production must be indicated; for food of animal origin, this is the farm where the animals were kept. Furthermore, the number of the control body for organic produce must also be displayed close to the EU organic logo [25].

### 2.3 Blockchain methodology

The central innovation behind the cryptocurrency Bitcoin is a technology that operates as a decentralized database, stored on many computers within a network [29]. Individual entries within that database are combined and stored in blocks that are chronologically concatenated (the blockchain) using cryptographic methods. When the blocks are created, a consensus must exist to ensure the authenticity of the database entries. To achieve consensus within a blockchain, all nodes must possess the rules with which the current consensus state was reached; importantly, each node can verify the state of consensus independently [30].

## 3. Results and discussion

### 3.1 Control of organic produce according to current EU legislation

In the EU, the legal basis for food safety is provided by the basic regulation (EC) No 178/2002 and the EU control regulation (EU) 2017/625. Overall, EU legislation grants individual member states the power to determine whether controls should be carried out by government agencies or by external providers supervised by the government. Germany, for example, operates a state-supervised private system in which the individual control bodies for organic produce are monitored by the competent state authorities in the individual federal states as the latter are responsible for the planning and control of official food monitoring [31]. In addition to national regulations, the requirements for the approval of private inspection bodies are specified by regulation (EU) 2017/625. To ensure the equivalence of organic produce control in all EU member states, all control bodies for organic produce must be accredited [32]. The control bodies are subject to constant supervision by the authorities and operate with an obligation to report to the competent authority, so that control competence and independence can be demonstrated on a regular basis [25].

All companies that produce, prepare, store or import organic products from a third country or enter these into the market under the scope of regulation (EU) No 2018/848 are subject to the control with the aim to establish an uninterrupted chain of control to prevent mixing of organic with conventional products and to ensure continuous traceability. Typically, a full site inspection takes place at least once a year and additional unannounced inspections occur according to a risk-based approach. Notably, companies that either sell unpackaged goods directly to the final consumer or do not exceed a certain annual sales volume are exempt from such controls [25].

During such controls, the existence of proper documentation regarding purchases, goods receipts, manufacturing processes and goods supply to downstream partners is examined and a plausibility check of the turnaround (flow of quantities) is done. Furthermore, production processes and storage practices are checked to determine whether accidental mixing or confusion with non-organic products can be ruled out. Upon completion, the control bodies issue organic farm certificates which attest that the company has subjected its activities to the control of organic produce and complies with the provisions of the EU legislation on organic produce. However, these certificates cannot provide unequivocal proof that an individual delivery is indeed organic produce. Usually, certificates are valid for one year or more and can be viewed on websites on a daily basis. Upon receipt of goods, companies must independently check the validity of their suppliers’ certificates [28].

In the event of violations, companies are penalized by a graduated catalogue of sanctions, which can lead up to a ban on the marketing of any products with organic labelling. Special provisions apply to imports of organic products from third countries [25].

### 3.2 Marketing standards in compliance with EU legislation

EU marketing standards as per the Directive 2002/4/EU require that eggs, unless sold directly at the production site, carry a producer code on the egg used to identify the farming method with a digit, followed by the origin member state (two letters) and the production company (combination of numbers). In some member states, a number for each producer’s farm and individual herds/stables are also assigned.

On the packaging, the production site, name and address of the production farm, country code, producer code, the number and/or weight of eggs, the laying date or period and the date of shipment needs to be specified. If the eggs are transported to a collection point or packing station in another EU member state, they must be marked with the producer code before leaving the production site (albeit exemptions are possible if the member state in which the packing station is located agrees and a copy of the supply contract is included). In addition, the packing station needs to be identified by a country code and packing station number; the code number of the inspection body for organic produce must be indicated.

### 3.3 Determinants of a theoretical traceability chain in compliance with EU organic legislation and egg marketing standards

**Fig 2:**
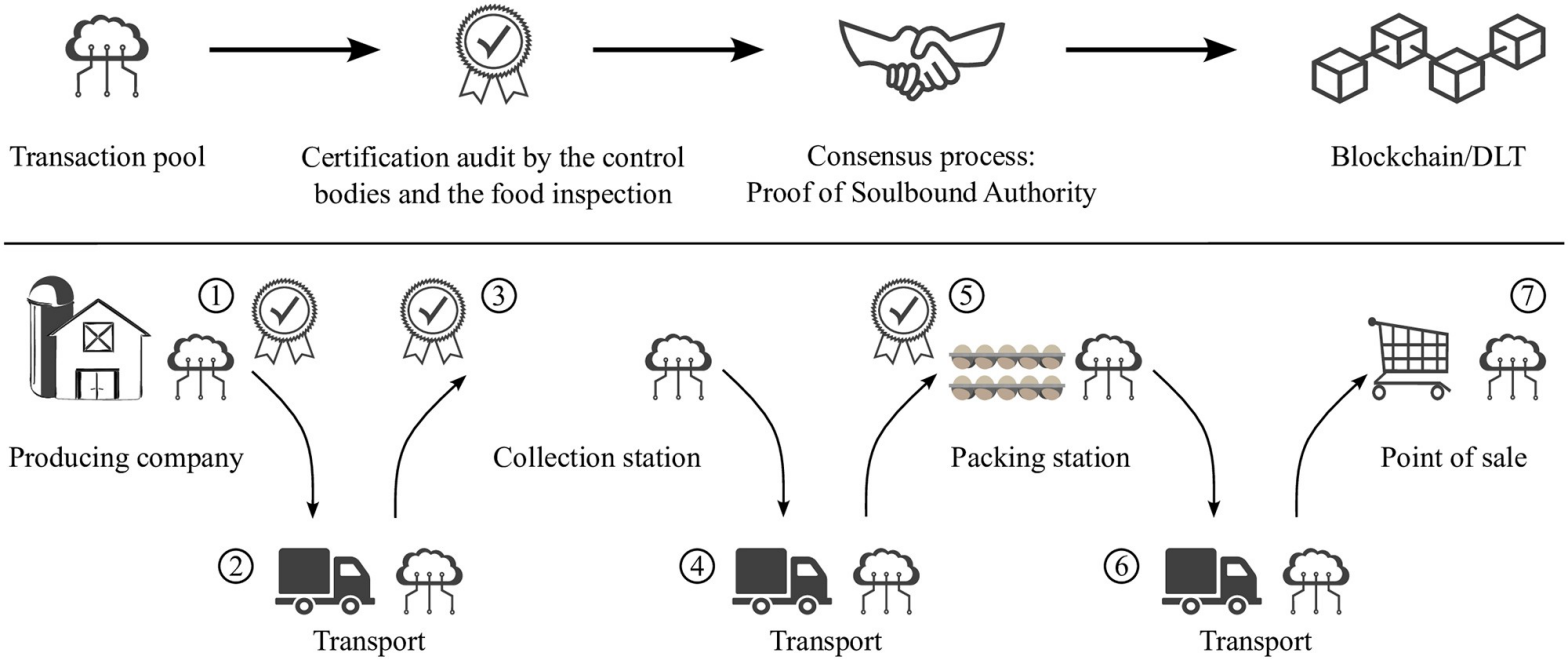
Theoretical design of a blockchain-based traceability chain for organically produced eggs. Upper panel: Steps involved in adding information to the blockchain. Transactions are added to the transaction pool and attain the status of ‘confirmed’ during the consensus process. Confirmed transactions are added to the blockchain. Lower panel: Schematic implementation of the blockchain concept in the egg supply chain. According to EU law, only units 1, 3 and 5 will possess certificates as these are issued by the inspection bodies for organic produce to any farm that produces, prepares, stores or imports organic products from a third country or places them on the market [25]. The purchasing retailer and the transport units, if these are independent entities, therefore have no certificates of their own that would need to be considered in the blockchain.

For the purpose of this study, we consider all participants to reside within the territory of the EU and therefore have not considered imports from third countries. Hence, when surveying the legal requirements, we restricted analyses to relevant secondary legislation (see S1 Table) dealing with production and control. A schematic illustration of a traceability chain for eggs is shown in Fig 2. Transactions take place between the individual links of the supply chain and are validated by certificate and consensus checking against a transaction pool. A transaction always involves two matching transaction proposals by the sending unit (units 1–6) and by the receiving unit (units 2–7). In order to allow complete traceability of the eggs, every physical transfer needs to be mapped as a transaction. There are thus seven units in total through which eggs can pass within the traceability chain (see Fig 2 bottom panel). If several links are processed within an individual unit, then the transports linking two subsequent units may be omitted. In such cases, a direct transaction between these two units can occur; this is possible between the point of production/collection point and collection point/packing point.

A full transaction therefore comprises two transaction proposals that agree in all of their parameters except the time stamp. The time stamp of the receiving transaction proposal needs to be at a later time than that of the transaction proposals by the sender. Within the consensus procedure, a pair of transaction proposals is only confirmed by the validator nodes, if they fulfil both of these criteria and, additionally, a valid certificate exists whenever units 1, 3 or 5 (see Fig 2) are involved. These certificates reside with the institution operating the validator nodes (control body for organic produce) and are issued by the official food control based on compliance with directive 2002/4/EC and the regulations (EU) No 589/2008, (EC) No 178/2002 and (EU) 2017/625. Upon confirmation of transactions, these are signed by the control body for organic produce and combined with other transactions into a block of the Blockchain. The legal framework stipulates that only the certificates issued by the official food control can freely be viewed by anyone. The resultant blockchain is thus a permissioned and private blockchain that allows tracing of transaction history only for the official food control and the control body for organic produce.

### 3.4 Implementation of certificates of operation in the blockchain traceability chain

Given the requirement that certificates validating an individual link of the supply chain need to be freely viewable by anyone (see above), soulbound tokens (SBTs) are best suited to map the real-world certificates in the traceability chain. SBTs are publicly viewable digital identity tokens that are non-transferable but revocable. They are issued by a higher to the next lower authority and represent the properties, characteristics and achievements of a person or identity. Token holders (‘souls’) can either issue SBTs to themselves or to other souls [33].

**Fig 3:**
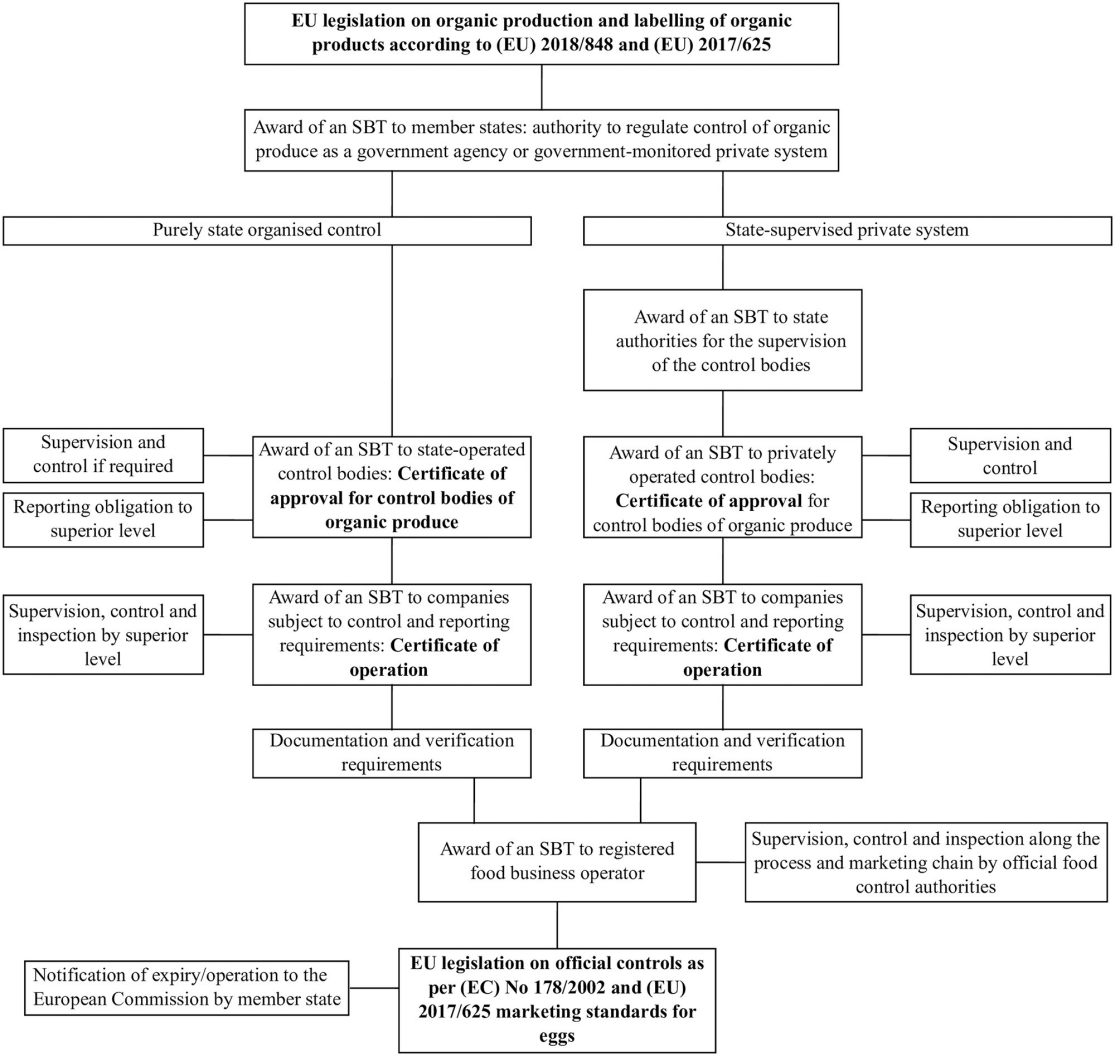
Issuing soulbound tokens (SBT) as certificates to regulate the status of each actor involved in control. The scheme shown assumes emission of soulbound tokens for control of organic produce by the European Commission to the member states as the top-level authority. This concept is in line with ongoing efforts surrounding the European Blockchain Services Infrastructure.

Fig 3 presents a suggested hierarchy network that maps the emission of certificates as well as the delegation of control to various entities within a blockchain-based traceability chain. The suggested hierarchy network conforms with the requirements by current EU legislation on the production and marketing of organic eggs, and the issuance of SBTs includes official controls in addition to the control of the status of organic production according to regulations (EC) No 178/2002, (EC) 2017/625 as well as the marketing standards.

The suggested framework allows the issuance of a certificate demonstrating the integrity of a laying hen farm after the farm has been registered according to Directive 2002/4/EC. Individual units fulfil all their reporting and documentation obligations electronically and the validity of SBTs/certificates is automatically checked when a transaction is processed within the blockchain traceability chain. A unit remains certified as long as all supervisory, control and documentation obligations are fulfilled. Via the validator nodes, the public can access the SBTs/certificates, but not any other documentation. In case of violations, two alternative mechanisms are possible: either the certificate is withdrawn or a certificate of deficiency is issued, which can be replaced by a regular certificate after the unit returns to compliance.

One of the least resource-intense and least costly options to establish agreement between the participants/nodes on the status of the distributed database (consensus mechanism) is the so-called proof of authority (PoA) that works by selecting its validators based on reputation. The identity of the validators must be unambiguously determined, checked against a reliable public database and subjected to an application process (i.e. control bodies for organic produce need to be approved by the governing body). Transactions are typically processed faster and more efficiently than with other consensus mechanisms, but this comes with a lesser degree of decentralization (due to the strict selection of validators) and requires higher levels of protection than other consensus mechanisms [34].

Given that a traceability chain for organically produced eggs in the EU requires a permissioned blockchain (see above) with only select entities (control nodes) participating, a PoA mechanism seems best suited. In combination with the use of SBTs, the resultant consensus mechanism becomes a ‘proof of soulbound authority’.

### 3.5 Automated real-time controls

Importantly, the point of entry of a batch of eggs into the traceability chain is of crucial significance, as the initial transaction assumes that the eggs can justifiably be classified as being organic produce. This initial property is then merely handed to any subsequent transactions. In order to provide means of automated control, sensor systems based on internet-of-things technology could be connected to the blockchain-based traceability chain and thereby provide a link between the real and virtual products in the chain. Individual transactions in the traceability chain can then be made subject to the chosen parameters meeting the necessary requirements. However, the choice of appropriate parameters to be monitored and automatically analyzed (e.g. video monitoring of husbandry, occupation density of stables, conformity with specifics of the free-range area, temperature of storage, etc) as well as the financial investments required for their implementation in the participating production sites and the storage and transport facilities may present a likely bottleneck.

However, a fully automated evaluation of the required documentation could be implemented in a fairly straightforward fashion if producers, collection points and packing stations document their data, evidence and other obligations electronically in a standardized form on a system accessible to the control body (as opposed to e.g. paper-based, in-house). Then, a continuous monitoring of the documentation within the blockchain consensus procedure is ensured and enables, for instance, an automated plausibility evaluation of the flow of quantities and, possibly, automated intervention by denial of a transaction.

### 3.6 Limits of automated intervention: Balancing penalty and incentives

The new EU legislation on organic produce stipulates consequences for either the presence of unauthorized substances or a suspicious indication without the presence of the same. However, if control body learns of unauthorized products and substances, a fully automated and mandatory program of action is triggered and a root cause investigation in the form of an official inquiry begins, along with a temporary ban on reselling or processing the products in question under the "organic" label.

In the case of an indication of a possible irregularity, various implications are conceivable. If the irregularity is noticed by the producer, they can themselves check whether there has been a breach of integrity; if there is no suspicion in this regard, the investigation is terminated. If suspicion persists and the root cause can be identified, it can be eliminated by the producer. Only if the suspicion cannot be eliminated, the control body needs to be informed immediately. However, a provisional removal of the "organic" label is not compulsory in this case. A fully automated transaction chain that includes self-governed trigger mechanisms based on monitored parameters presents a rigid mechanism that would deny transactions fairly quickly under such circumstances, and possibly lead to a system that has neither been the intention of the EU legislation nor meet the realities of the producers, thus impeding the production of organic produce overall rather than fostering it. This concern is supported by a modelling study probing the impact of various blockchain parameters on the behavior of the various stakeholders of a traceability chain. Importantly, the introduction of positive incentives into such a system as well as keeping the punishment moderate was found to deliver the optimal behavior with respect to share true information. Highly severe punishments (such as e.g. the quick removal of the “organic” label as outlined above) resulted in low willingness to share initial true information [35].

## 4. Conclusions

Clearly, a blockchain solution backing a traceability chain would offer a number of practical benefits. During controls, a comparison of data could happen automatically and at any time, if the obligatory documentation was stored in a system to which the control body has access. Thereby, a fully automated plausibility check of the quantity flows could be carried out.

At the current state of matter, however, the core property of the eggs entering the system as organic produce rests on the fact that their status is indeed ‘organic’ in the initial transaction. This initial status is simply passed on in all subsequent validated transactions. Whereas IoT technology could be used in principle to augment the information collected at the laying hen farm, the overall egg production is a complex process involving farming conditions, feed and potentially other factors, thus rendering a full coverage of all involved production aspects by IoT infeasible. This results in a degree of disconnect between individual produce items and the documentation and information entered into the blockchain. Therefore, a blockchain solution for traceability of organically produced eggs seems unlikely to be implemented as an entirely self-governed system. The validation of the state of the eggs will require regular controls of the production facility and random sample checking (anywhere in the chain) by analytical methods that are being developed [36, 37].

Governance in such a solution would therefore occur by the temporary or permanent revocation of certificates by control bodies if either a site inspection on the farm or results obtained from analytical investigation of samples yields evidence of non-compliance with the requirements for organic produce.

The implementation of such a solution as a non-self-governed system and the resultant governance/intervention mechanisms would retain some scope for action for the producer to eliminate reasons for perceived anomalies or refute suspicions of non-compliance. A fully self-governed blockchain solution that considers all EU legislation on organic produce, in contrast, could potentially result in an immediate revocation of the certification of a producer if a control node reports the perceived presence of unauthorized products or substances. For the latter situation, EU legislation requires the start of a mandatory action program and root cause inspection during which all sales or processing of the eggs labelled as organic produce must be suspended.

The traceability chain presented here, like the overarching EU legislation, has a limitation in not providing unambiguous proof of the organic status of individual products. However, the concept proposed offers advantages with respect to allocation of authority at EU level and might have positive effects beyond the traceability chain. Particularly, it allows development of a system of incentives that can lead to increased self-regulation, while recognizing the importance of EU control and its role in ensuring compliance with existing and future rules and policies.

While this research primarily presents a theoretical framework for the integration of blockchain technology in the traceability of organic egg production in the EU, it is acknowledged that the transition from theory to practice presents significant challenges. Future research could focus on developing pilot projects or case studies in collaboration with organic egg producers and blockchain technology providers. These practical implementations would not only test the feasibility of the proposed solution but also provide valuable insights into operational challenges and the effectiveness of the regulatory framework in real-world scenarios. Additionally, forming partnerships with relevant stakeholders in the organic egg supply chain could facilitate the exchange of knowledge and foster an environment conducive to innovation and practical application of this technology.

## Supporting information

S1 Table Legal framework of the new EU legislation on organic produce as of July 2022. (PDF)
