# Supplementary material for: Theoretical design of blockchain-based traceability for organic egg supply chains according to regulation (EU) 2018/848
Source: PLoS One. 2024 Jun 11;19(6):e0304791. doi: 10.1371/journal.pone.0304791 (PMC11166310; doi:10.1371/journal.pone.0304791)
Supplement: S1 Table — (PDF) [file pone.0304791.s001.pdf]

# Supplementary Information

*Table S1 Overview of the legal framework of the new EU legislation on organic produce as of July 2022.DA: delegated act, IA: implementing act.*

| Legal bases | | |
|---|---|---|
| **Regulation (EU) 2018/848**<br>EU rules on producing and labelling organic products | | |
| **Regulation (EU) 2017/625**<br>Requirements for the implementation of controls of organic produce | | |
| **Secondary legislation supplementing the EU legislation on organic produce** | | |
| **Production** | **Control** | **Imports from third countries** |
| **Regulation (EU) 2020/464**<br>IA: Animal husbandry, processing, availability databases, conversion | **Regulation (EU) 2021/279**<br>IA: Contaminations, group certification, control quotas, traceability, catalog of measures | **Regulation (EU) 2021/1698**<br>DA: Third country regulations: Admission procedures, monitoring, control rules, reports, etc |
| **Regulation (EU) 2020/2146**<br>DA: Exceptions in the event of disasters | **Regulation (EU) 2021/1006**<br>DA: Organic certificate (template change) | **Regulation (EU) 2021/1697**<br>DA: Modification of the criteria for the recognition of control |
| **Regulation (EU) 2021/1189**<br>DA: Ecological heterogeneous material | **VO (EU) 2021/771**<br>DA: Traceability check, mass balances (flow of goods), group certification | **Regulation (EU) 2021/1342**<br>DA: Monitoring and verification of equivalent third countries/control bodies |
| **Regulation (EU) 2021/1165**<br>IA: Annexes Operating materials, additives, processing aids | **Regulation (EU) 2021/2119**<br>IA: Company records | **Regulation (EU) 2021/1378**<br>IA: Organic certificates for third country imports, list of inspection bodies |
| **Regulation (EU) 2020/427**<br>DA: Changes to sprouts, feeding bees, young animals and feeding aquaculture | **Regulation (EU) 2021/715**<br>DA: Group certification company (changed requirements) | **Regulation (EU) 2021/2306**<br>DA: Inspection certificate, border inspection posts |
| **Regulation (EU) 2020/1794**<br>DA: Amendments to conversion and non-organic plant propagating material | **Regulation (EU) 2021/1691**<br>DA: Change recording obligations of companies | **Regulation (EU) 2021/2307**<br>IA: Customs declarations, partial inspection certificate via *TRACES* |
| **Regulation (EU) 2021/642**<br>DA: Changes labeling compound feed and seed mixtures | | **Regulation (EU) 2021/2304**<br>DA: Export certificate antibiotics |
| **Regulation (EU) 2021/716**<br>DA: Changes to sprout production, chicory and parasite treatments (aquaculture) | | **Regulation (EU) 2021/2305**<br>DA: Exempt from official controls at border inspection posts, supplement to regulation (EU) 2017/625 |
| | | **Regulation (EU) 2021/2325**<br>DA: List of equivalent third countries and inspection bodies for imports |
